# Type 2 cytokine genes as allergic asthma risk factors after viral bronchiolitis in early childhood

**DOI:** 10.3389/fimmu.2022.1054119

**Published:** 2023-01-06

**Authors:** Zihan Dong, Åsne Myklebust, Ingvild Bjellmo Johnsen, Tuomas Jartti, Henrik Døllner, Kari Risnes, Andrew T. DeWan

**Affiliations:** ^1^ Department of Biostatistics, Yale School of Public Health, New Haven, CT, United States; ^2^ Center for Perinatal, Pediatric and Environmental Epidemiology, Yale School of Public Health, New Haven, CT, United States; ^3^ Children’s Clinic, St Olav Hospital, University Hospital, Trondheim, Norway; ^4^ Department of Clinical and Molecular Medicine, Norwegian University of Science and Technology, Trondheim, Norway; ^5^ PEDEGO Research Unit, University of Oulu, Oulu, Finland; ^6^ Department of Pediatrics and Adolescent Medicine, Oulu University Hospital, Oulu, Finland; ^7^ Department of Pediatrics and Adolescent Medicine, Turku University Hospital and University of Turku, Turku, Finland; ^8^ Department of Chronic Disease Epidemiology, Yale School of Public Health, New Haven, CT, United States

**Keywords:** childhood asthma, viral respiratory infection, bronchiolitis, type 2 cytokines, genetic risk factors, gene-environment interaction (G × E)

## Abstract

**Background:**

Genome-wide association studies of asthma have identified associations with variants in type-2 related genes. Also, specific interactions between genetic variants and viral bronchiolitis in the development of asthma has been suggested.

**Objective:**

To conduct a gene-based analysis of genetic variants in type 2 cytokine related genes as risk factors for allergic asthma at school age, and further, to study their interaction with specific viral infections in early childhood.

**Methods:**

A prospectively investigated cohort of children with previous bronchiolitis and controls came for follow-up at school age. The research visit, blinded to viral exposure, included detailed lung function tests, laboratory investigation, and questionnaires. Allergic asthma was defined as typical symptoms plus objective variable airway obstruction, in addition to laboratory verified atopy (elevated eosinophil count or sensitization to an allergen). Targeted and complete sequencing was performed for nine type 2 cytokine candidate genes: IL4, 5, 13, 25, 33 and 37, IL17RB, CRLF2 and TSLP.

**Results:**

At follow-up, there were 109 children with genetic data, 91 with a history of bronchiolitis (46% respiratory syncytial virus, 24% human rhinovirus, 15% human metapneumovirus and 14% mixed viral etiology) and 18 without. The median age was 9.4 years (range 6-13) and 41 (38%) had laboratory verified atopy. Twenty-one children (19%) met the definition of allergic asthma. After adjusting for age, sex and five viral categories, IL33 achieved nominal significance (p = 0.017) for a positive association with allergic asthma development. In the gene-virus interaction analysis, the variant set in IL17RB demonstrated a nominally significant positive interaction with human metapneumovirus infection (p=0.05).

**Conclusion:**

The results highlight the multifactorial nature of allergic asthma risk, with both viral infection and inherited genetic variants contributing to increasing risk. Results for IL33 and IL17RB were nominally significant and are potential candidate targets for designing therapeutics and early screening, but these results must be replicated in an independent study.

## Introduction

Asthma is the most common chronic disease among children, and is characterized by airway hyperresponsiveness, mucus hypersecretion, and an exaggerated inflammatory response usually dominated by type 2 cytokines (1). The possible role of inherited genetic risk variants related to type 2 cytokines and asthma development following viral bronchiolitis has not been considered in previous studies. There is also evidence that virus-induced bronchiolitis is associated with recurrent wheezing and asthma, particularly rhinovirus, that has been recognized as an important risk factor for asthma (2–4).

IL33 and thymic stromal lymphopoietin (TSLP) are cytokines functioning as alarmins that are released by the airway epithelium in response to viruses, allergens and other triggers and drive type 2 responses in asthma. IL33 and TSLP both have synergistic effects on type 2 cytokines production and eosinophil levels (5, 6). TSLP and IL33 may be important new targets individualized for asthma treatment, and antibody-based drugs for these pathways are being developed (7, 8). Tezepelumab is an anti-TSLP human monoclonal antibody that normalizes type 2 cytokine levels (9) and in clinical studies significantly reduced exacerbation rates vs placebo in patients with severe, uncontrolled asthma (10). Astegolimab is an anti-ST2 antibody that blocks IL33 signaling and has shown promise in reducing asthma exacerbations (11). Airway epithelial cells (AECs) from humans with asthma have increased TSLP mRNA levels (12), and over-expression of TSLP induces experimental asthma in mice (13). Rhinovirus (RV) infection in young children was found to be associated with elevated levels of TSLP (14). Further, respiratory syncytial virus (RSV) and metapneumovirus (MPV) infection induce production of TSLP in AECs *in vitro* and *in vivo*, respectively (15, 16).

IL25 also triggers expression of type 2 cytokines, activates type 2 innate lymphoid cells (ILC2 cells) and induces allergic asthma, all of which is dependent on its receptor, IL17RB (17–19). As with TSLP, overexpression of IL25 can induce asthma-like disease in mice (20, 21). Further, RV infected epithelial cells cultured from asthmatic patients have a higher capacity to produce IL25 (22). Using an established mouse model of allergic airway inflammation, infection with rhinovirus led to an increased production of IL25 as well as increased infiltration of eosinophils, neutrophils and basophils, secretion of mucus and production of type 2 cytokines. Importantly, these effects were neutralized if the animals were treated with an antibody directed against IL17RB (22).

While genome-wide association studies of asthma have identified associations with variants in genes that encode cytokines, cytokine receptors and related proteins [e.g. IL13, IL4, IL33, IL1RL1, IL1RL2, IL18R1, TSLP; see Demanais et al. (23)], an examination of genetic variants related to excessive type 2 cytokine production and their interactions with viral infections may shed insight into specific asthma mechanisms. Here we conduct a gene-based analysis of variants in type 2 cytokine related genes (i.e. IL4, 5, 13, 25, 33 and 37, IL17RB, CRLF2 and TSLP) as risk factors for allergic asthma at school age and study their interaction with specific viral infections in early childhood (**Table 1**). We hypothesized that variants in type 2 cytokine related genes are associated with the susceptibility to virus-induced early wheezing and development of allergic asthma at school age, and that such associations may show interactions with specific viral infections.

**Table 1:** Description of the selected candidate genes and their role in type 2 responses.

Gene	Function	Main role in type 2 responses in lung
IL4	Cytokine	Activation macrophage type 2, ILC2 (24)
IL5	Cytokine	Induces eosinophilia (24)
IL13	Cytokine	Activation of macrophage type 2, fibrosis, mucus production, IgE isotype switch (24)
IL17RB	Receptor for IL-25	Activated by IL25 (17–19)
IL25	Cytokine/alarmin	Triggers type 2 cytokine expression, activates ILC2 cells, induces allergic asthma. Induces asthma in mice (17–21)
IL33	Cytokine/alarmin	Triggers type 2 cytokine expression and eosinophil levels (5, 6) and IL 33 levels corelate with asthma disease severity (25)
IL37	Regulatory cytokine (IL-1 family)	Inhibition of Th1/Th2/Th17 inflammatory mediators. Suppress allergic inflammation in asthma by suppression of innate and acquired immunity (26, 27)
CRLF2	Receptor for TSLP	Forms a complex with TSLP and stimulates type 2 inflammatory proliferation and differentiation (28)
TSLP	Cytokine/alarmin	TSLP levels correlate with asthma severity (12) and induces type 2 cytokine production and eosinophil levels, synergism with IL-33 (5, 6)

## Methods

### Study subjects

The study population was recruited from a prospective, regional, population-based surveillance cohort of children admitted for airway infections (The St Olav Hospital Airway Project Cohort) (29–31). Eligible participants for the present follow-up study had initially been hospitalized between 2006 and 2012 and had nasopharyngeal secretion (NPS) routinely tested with in-house polymerase chain reaction (PCR) for 17 viruses. The inclusion criteria for the follow-up study were lower respiratory tract infection before 2 years of life with referral to pediatric specialist care, or children from the original control group consisting of surgery patients and without lower viral infection exposure (see Main Effects section for details on how viral infections were classified). All journals were revised after the research visit, and two subjects were excluded because of bacterial pneumonia at exposure.

The study was approved by the Regional Committee on Medical Research Ethics (REK number 2016/540). Informed consent for the clinical follow-up at school age was collected and included consent for the genetic analyses.

### Study protocol

The data from the hospitalization was prospectively collected (29–31). The follow up after 6 years of life was systematically set up for research and was not part of routine practice. Examination of the children and data collection took place at the Research Facility Ward at St Olav Hospital between March 2017 and June 2019 and was led by a pediatric asthma specialist together with a trained research nurse, both blinded for viral exposure. The research visit included lung function testing, blood sample draw after local anesthesia, a systematic medical history and clinical exam by the study pediatrician. A digital questionnaire for care givers was based on The International Study of Asthma and Allergies in Childhood Questionnaires (ISAAC) (32) related to history of asthma and atopy in childhood.

### Lung function testing

Lung function was measured according to established guidelines (33, 34) using a Vyntus Pneumo APS spirometer. The results were obtained both as absolute values and in % of predicted values according to EU standard data for age, height and sex. After initial baseline flow-volume spirometry attempts all children were considered for a methacholine provocation test (MPT) to measure eventual bronchial hyper-reactivity. Contraindications for the MPT was any airway infection, ongoing antibiotic treatment or asthma exacerbation during the last two weeks, FEV_1_ <70%, clinical signs of airway obstruction, technical inability to perform reproducible and repeated spirometries. The MPT test was performed with an inhalation-synchronized dosimetry nebulizer. The test procedure implicated doubling of doses of methacholine administered until a 20% fall in FEV_1_ or to a maximum cumulative dose of 1,447 mg methacholine. All children concluded the lung function reversibility testing with a spirometry after inhalation of salbutamol 0.4 mg (Ventoline) through a spacer (Optichamber Diamond by Philips Respironics).

### Phenotype definition

The allergic asthma diagnosis required both a clinical diagnosis of asthma based on recent guidelines and one of the laboratory criteria (35). Clinical asthma was diagnosed if either 1) presence of one or more typical asthma symptoms in history plus variable expiratory airflow limitation at study visit or 2) recent asthma diagnosed by a pediatric specialist and ongoing treatment with inhaled corticosteroids at research visit. Asthma symptoms were defined as cough at night or prolonged cough > 14 days during viral infections, exercise induced either chest tightness, wheeze or shortness of breath, or recognition of these symptoms during methacholine testing when inducing airflow limitation. Variable airflow expiratory limitation, or reversibility, was defined as either a fall in FEV_1_ of ≥ 20% during the metacholine challenge, or for those who only performed spirometry an increase of FEV_1_ ≥ 12% compared to the baseline spirometry after salbutamol inhalation. The laboratory criteria were defined as either allergic sensitization (specific IgE > 0.35 kU/L to any of the tested 11 aeroallergens or 6 food allergens) or blood eosinophil count≥300 cells/µL.

### Sequencing

Sequence data was generated for 111 of these subjects. Sequencing was done for nine candidate genes (i.e. IL4, 5, 13, 25, 33 and 37, IL17RB, CRLF2 and TSLP) to capture all exons, intron/exon boundaries and up- and down-stream regulatory regions. Library prep was conducted using AmpliSeq for Illumina custom DNA panel (Illumina Inc., CA), which contained 203 amplicons (in two pools) with average amplicon length of 244 bp and covered in total 28,546 base pairs (27,076 covered bases). In brief, 5 ng DNA was used as input in two target PCR amplification reactions (one of each amplicon pool) of 18 cycles (99°C for 15 sec and 60°C for 4 minutes). Secondly, FuPa reagent was used to digest primer dimers and partially digest amplicons. In the next step dual sample indexes were added. The amplicon products were then cleaned up by using Mag-Bind® TotalPure NGS (Omega Bio-tek, Inc., Georgia, GA, USA) to purify the specific amplicons away from free primers and primer dimer species. A second amplification step which amplifies the libraries to ensure sufficient quantity for sequencing on the Illumina platform were then performed, prior to a second clean up by using Mag-Bind® TotalPure NGS (Omega Bio-tek, Inc., Georgia, GA, USA). Finally, a validation of the libraries was performed using an Agilent High Sensitivity DNA Kit on a Bioanalyzer (Agilent Technologies, Santa Clara, CA, USA).

Libraries were normalized and pooled to 8 pM and subjected to clustering on two MiSeq V3 flowcell. Finally, paired end read sequencing was performed for 2X150 cycles on a MiSeq instrument (Illumina, Inc. San Diego, CA, USA), according to the ‘manufacturer’s instructions. Base calling was done on the MiSeq instrument by RTA v1.18.54. FASTQ files were generated using bcl2fastq2 conversion software v2.17 (Illumina, Inc. San Diego, CA, USA).

### Sequence data alignment and quality filtering

Sequencing adapters and low-quality reads were removed using Atropos (v. 1.1.18, flags -q 15 and -m 15). The processed reads were aligned against the human genome (hg38) using BWA MEM (v. 0.7.15, flags -M and -v 2). The BAM files were sorted and read group (RG) information was added, followed by cleaning, soft-clipping, and marking of duplicate reads using AddOrReplaceReadGroups, CleanSam, and MarkDuplicates (OPTICAL_DUPLICATE_PIXEL_DISTANCE = 2500, CREATE_INDEX = TRUE) from Picard tools (v. 2.18.5). Base quality scores were recalibrated using GATK’s (v. 4.0.4.0) BaseRecalibrator and ApplyBQSR based on known sites of variation (dbSNP v. 151 and Mills and 1000G gold standard from GATK’s resource bundle). Variants were then called for each sample separately using GATK’s HaplotypeCaller, before merging all resulting gvcf files with GATK’s CombineGVCFs and calling variants on the joint file using GATK’s GenotypeGVCFs (flag -G StandardAnnotation). Called SNVs and indels were processed separately to filter high quality variants using ‘GATK’s VariantFiltration, keeping SNVs with QD < 2.0, FS > 60.0, MQ < 40.0, MQRankSum < -12.5, and ReadPosRankSum < -8.0, and indels with QD < 2.0, FS > 200.0, and ReadPosRankSum < -20.0.

### Subject quality control

Following variant calling and initial quality control, we examined the per subject call rate to determine if there were any subjects with an indication of low-quality data across all variants. All included subjects (N=109) had a call rate across the 350 variants > 85% and therefore all were included in the analysis.

### SNP quality control and annotation

Among all variants identified by sequencing, 350 were annotated to the nine genes by UCSC GTEx Gene V8 (within 20kb of the gene start and stop site). We excluded variants with call rate < 90% (N=41) and then with a Hardy–Weinberg equilibrium P-values < 10^-4^ (N=5) leaving 304 variants for analysis. The included variants were annotated by ANNOVAR (36) for functional annotation (**Supplementary Table S1**).

### Main effects

Our main analysis examined the association with allergic asthma case/controls status. Gene-based analysis integrates information across all included SNPs within one gene or genomic region to improve the statistical power of the association analysis. The gene-based analysis was conducted using SKAT (37). with all parameters being default values. When the sample size is less than 2000, SKAT automatically applies small sample size adjustment. Missing genotype values are imputed based on Hardy-Weinberg equilibrium for binary outcomes. Covariates include age and sex. Viral infections were classified into five categories: 1) sole RSV infection; 2) sole RV infection; 3) sole MPV infection or in combination with viruses other than RSV, RV or bocavirus (BoV); 4) mixed infection (co-infections including RSV, RV, MPV or BoV and any other virus); 5) no viral infection. Dummy coding was utilized to adjust for viral infections in the analysis. Study-wise statistical significance was determined by Bonferroni correction for the nine genes at alpha = 0.05 (p<0.05/9 = 0.0056) and nominal significance was considered at p <= 0.05.

SNP-level association tests were conducted using command “SKATBinary_Single” and p-values were computed. We ran logistic regression to calculate the direction of effect of each SNP.

### Sensitivity analyses

Given the small sample size, we conducted several sensitivity analyses to test the robustness of our results to various parameters: 1) we applied the SKAT-O (38) method instead of SKAT since SKAT-O combines both the variance components and burden tests; 2) we analyzed only low-frequency variants with a minor MAF < 5% and 3) instead of dummy-coding the five indicator variables representing each viral category and adjusting for each, we merged this into one dichotomous covariate which indicates whether (viral categories 1-4) or not (viral category 5) an individual had been exposed to viral bronchiolitis.

### Interaction analysis

We tested the interaction between the specific virus types, rhinovirus, RSV and MPV, and each of the nine gene-based variant sets using the R package “iSKAT” (39). We utilized the same categorization for rhinovirus, RSV, MPV described previously and adjustment for the other virus categories not contained in the interaction was done using dummy coding. In addition, age and sex were included as covariates in each model. All parameters were default values.

## Results

There were 135 participants who came to the follow-up visit, 109 of these children were available for genetic analysis. **Table 2** shows their clinical characteristics. The age at follow-up ranged from 6 – 13 years and there were 41 females (38%). There were 42 from the RSV group, 22 from the RV group, 14 from the MPV group, 13 from the mixed infection group and 18 with no bronchiolitis exposure. Twenty-one children (19%) met the definition for allergic asthma, all of them corresponded to mild asthma. As expected children with RV bronchiolitis exposure were more likely to have allergic asthma (29%) than not (18%), the opposite was seen for the RSV group (19% with allergic asthma vs 43% without).

**Table 2:** Clinical characteristics study subjects.

Characteristics	Allergic asthma at follow-up (%^1^)	Controls (%^1^)
Total	21	88
Age [years, IQ-range]	9.1 (8.0 -11.1)	9.5 (8.5 – 10.8)
Female	11 (52)	31 (35)
Parental asthma or allergy	18 (86)	57 (65)
Sensitization or eosinophilia	21 (100)	20 (23)
Virus etiology^2^		
RSV^3^	4 (19)	38 (43)
RV^4^	6 (29)	16 (18)
MPV^5^	4 (19)	10 (11)
Mixed infection^6^	4 (19)	9 (10)
no respiratory tract infection exposure^7^	3 (14)	15 (17)

^1^Unless otherwise noted.

^2^For the four virus positive categories, these subjects were hospitalized with respiratory tract infection with viral etiology based on PCR on nasopharyngeal samples (31).

^3^RSV: sole RSV infection.

^4^RV: sole RV infection.

^5^MPV: sole MPV infection or in combination with viruses other than RSV, RV or BoV.

^6^Mixed infection: co-infections including RSV, RV, MPV or BoV and any other virus.

^7^no viral infection.

Targeted sequencing of nine candidate genes was performed to identify all potential variants in each gene (N=350 variants with 304 that passed quality control [see Methods]). Using all coding variants annotated by ANNOVAR (36) to each of the nine genes, aggregate gene-based analyses were conducted for each of the variant sets. We examined the association between each of the nine gene-based aggregate of variants with allergic asthma case/controls status. After adjusting for age, sex and the five viral categories, IL33 achieved nominal significance (P = 0.017, **Table 3**). Among the samples, IL33 contained 37 coding variants, but none of the single variants achieved statistical significance after adjusting for all 304 variants but five achieved nominal significance (p<0.05), all of which showed an association between the minor allele and increased risk of allergic asthma (**Table 4**).

**Table 3:** Gene-based association results using SKAT.

Gene^1^	P-value	Variants (N)	MAC^2^	m^3^
IL37	0.850	18	362	109
IL17RB	0.229	23	414	98
TSLP	0.425	47	686	102
IL5	0.293	8	24	18
IL13	0.457	12	184	65
IL4	0.158	6	120	34
**IL33**	**0.017**	**37**	**674**	**102**
IL25	0.898	19	263	71
CRLF2	0.094	26	121	78

^1^Genes with a nominally significant (p<0.05) p-value are indicated in bold.

^2^MAC: total minor allele count.

^3^m: number of subjects with minor allele(s).

**Table 4:** Single variant results for IL33.

Variant^1^	Chr^2^	Pos^3^	Gene^4^	P-value^5^	MAF^6^	Direction^7^	Annotation^8^
rs555998964	9	6215178	IL33	0.229	0.005	+	UTR5
rs746808236	9	6215231	IL33	0.608	0.005	–	intronic
rs1431798023	9	6215727	IL33	0.125	0.005	+	intronic
rs17498168	9	6237186	IL33	0.211	0.028	–	intronic
NA	9	6250936	IL33	0.496	0.037	–	intronic
rs1317230	9	6251012	IL33	0.205	0.317	–	intronic
**rs73398552**	**9**	**6252689**	**IL33**	**0.039**	**0.041**	**+**	**intronic**
rs149045797	9	6252690	IL33	0.535	0.005	–	intronic
rs1397714619	9	6252710	IL33	0.142	0.005	+	intronic
NA	9	6252720	IL33	0.142	0.005	+	intronic
rs10975519	9	6253571	IL33	0.861	0.367	+	exonic (synonymous)
rs562550122	9	6253690	IL33	0.068	0.005	+	intronic
rs10975520	9	6253710	IL33	0.660	0.358	+	intronic
NA	9	6255774	IL33	0.626	0.005	–	intronic
NA	9	6255781	IL33	0.626	0.005	–	intronic
rs12336076	9	6255789	IL33	0.602	0.321	–	intronic
NA	9	6255807	IL33	0.600	0.239	–	intronic
NA	9	6255808	IL33	0.785	0.028	–	intronic
NA	9	6255814	IL33	0.784	0.028	–	.
rs1013300156	9	6255821	IL33	0.819	0.133	+	intronic
NA	9	6255826	IL33	0.870	0.101	–	intronic
rs1332290	9	6255881	IL33	0.403	0.417	+	intronic
rs146597587	9	6255967	IL33	0.535	0.005	–	splicing
rs1240440599	9	6256010	IL33	0.732	0.005	–	exonic (synonymous)
NA	9	6256013	IL33	0.732	0.005	–	exonic (synonymous)
rs35375147	9	6256078	IL33	0.344	0.014	–	exonic (synonymous)
rs1048274	9	6256292	IL33	0.835	0.372	–	UTR3
NA	9	6256471	IL33	0.542	0.050	–	UTR3
NA	9	6256476	IL33	0.789	0.023	+	UTR3
**rs55726619**	**9**	**6256678**	**IL33**	**0.039**	**0.041**	**+**	**UTR3**
**NA**	**9**	**6256741**	**IL33**	**0.035**	**0.005**	**+**	**UTR3**
**rs12000491**	**9**	**6257367**	**IL33**	**0.039**	**0.041**	**+**	**UTR3**
rs189961633	9	6257571	IL33	0.787	0.009	+	UTR3
NA	9	6257597	IL33	0.099	0.014	+	UTR3
rs553100713	9	6257606	IL33	0.068	0.009	+	UTR3
NA	9	6257718	IL33	0.607	0.005	–	UTR3
**rs73398574**	**9**	**6257724**	**IL33**	**0.039**	**0.041**	**+**	**UTR3**

^1^Variant identification number (rsID), if assigned. Nominally significant associations with allergic asthma are presented in bold.

^2^Chromosome.

^3^Base pair position on the chromosome, in hg38 coordinates.

^4^Gene in which the variant was identified.

^5^P-value from the SKAT test.

^6^Minor Allele Frequency.

^7^Direction of effect estimated using logistic regression, where “+” represents a variant with a minor allele that has a higher frequency among asthmatic children and “-” represents a variant with a minor allele that has a lower frequency among asthmatic children.

^8^Functional annotation according to refGene; UTR3 = 3’ untranslated region; UTR5 = 5’ untranslated region.

To test if the IL33 gene-based association was robust to different inclusion/exclusion criteria for variants, subjects and analysis method, we performed a series of sensitivity analyses (**Table 5**). All sensitivity analyses were nominally significant indicating that the results are robust to these inclusion/exclusion criteria. Further, restricting to low frequency variants (MAF < 5%) yielded the same p-value (p=0.017), indicating that low frequency variants are driving the signal in IL33 rather than common variants.

**Table 5:** Sensitivity analysis results (P-values).

Gene^1^	SKAT	SKAT-O	MAF<5% (M = 210)	Virus/no-virus covariate coding
IL37	0.850	0.504	0.882	0.901
IL17RB	0.229	0.376	0.231	0.129
TSLP	0.425	0.638	0.547	0.422
IL5	0.293	0.428	0.293	0.238
IL13	0.457	0.520	0.563	0.682
IL4	0.158	0.205	0.120	0.144
**IL33**	**0.017**	**0.020**	**0.017**	**0.042**
IL25	0.898	0.104	0.952	0.949
CRLF2	0.094	0.172	0.084	0.171

^1^Genes with a nominally significant interaction p-value are indicated in bold along with the p-value for the respective tests.

We conducted a gene-virus analysis to identify potential interactions with specific viral types that may be interacting with the gene-based variant sets to alter the risk for asthma. The variant set in IL17RB demonstrated a nominally significant interaction with MPV infection (p=0.050; **Table 6**).

**Table 6:** Gene x virus interaction analysis results (P-values).

Gene^1^	RV	RSV	MPV
IL37	0.694	0.065	0.450
**IL17RB**	**0.510**	**0.846**	**0.050**
TSLP	0.631	0.884	0.201
IL5	0.612	NA^2^	0.241
IL13	0.146	0.822	0.506
IL4	0.622	0.186	0.814
IL33	0.490	0.672	0.370
IL25	0.076	0.097	0.766
CRLF2	0.545	0.510	0.258

^1^A gene(s) with a nominally significant interaction p-value are indicated in bold along with the p-value for the respective test(s).

^2^NA: Due to the fact that no subject with a rare variant in IL5 was also positive for an RSV infection the interaction matrix was 0 across all subjects and thus the interaction analysis was unable to be performed.

## Discussion

Using children from a well described bronchiolitis cohort and their controls that collected detailed phenotype and clinical data at school-age, we were able to detect a nominal association with asthma for the gene IL33. Further, we were able to detect a nominally significant interaction with MPV infection and variants in IL17RB despite a small sample size. These are promising findings that will need to be confirmed in a larger study sample and contribute to the growing literature of gene-virus interactions contributing to asthma development.

Type 2 cytokine hyperresponsiveness is a hallmark of both asthma and bronchiolitis following rhinovirus (40), RSV (41) and MPV (42, 43) infections. Therefore, an examination of variants within the genes that code for cytokines and their receptors is warranted to identify their potential role in interactions with viral infection and asthma risk. Previous studies identified an association between asthma susceptibility and a variant in IL17RB (44) as well as IL33 which was also associated across diverse ancestries (45, 46).

IL33 induces expression of a number of Th2 cytokines and thus increases eosinophilic inflammation. A rare loss of function variant in IL33 has been shown to be protective against asthma (8). While our analysis did not include any loss of function variants, this does further demonstrate the potential importance of low frequency and rare variants in the etiology of asthma. Variants included in our analysis of IL33 both increased and decreased risk for asthma development (**Table 4**), however, we do not yet know if the minor alleles increase or decrease expression of IL33 in relevant tissues following viral exposure.

IL17RB encodes the cytokine receptor interleukin-17 receptor B, a receptor specific for IL17B and IL25 (IL17E). IL17 knock-out mice appear to have a lower inflammatory response following MPV infection (42). Further, there is evidence of an intronic IL17RB variant (+5561G>A) in which the minor allele is protective against asthma and associated with lower IL17RB expression (44). Taken together, these observations suggest that genetic variations that increase IL17RB expression may increase the risk for asthma, particularly in those exposed to respiratory infections.

Unlike previous studies of gene-virus interactions for asthma, we chose a sequence-based approach to identify all variants in the coding regions of our selected candidate genes. Our primarily analyses aggregated variants across all those annotated to each gene rather than examining them individually. Our results for IL33 demonstrate that while several single variants within this gene were nominally associated with asthma, no single variant achieved the same level of statistical significance as they did in aggregate. By using a variance-components based method to analyze our aggregated variants, we increased our power to detect associations since the directions of effect of the variants in the IL33 gene were relatively evenly split between risk and protective. Further, our sensitivity results show that the gene-based association between IL33 and asthma is driven by low frequency variants (MAF <5%) which we had no power to detect associations with individually.

Unfortunately, we do not detect any association signals between allergic asthma and the remaining eight candidate genes, despite all of them being strong candidate genes for this phenotype. This could be due to out sequencing strategy of focusing primarily on coding and regulatory regions which may miss many of the tagging genetic variants that were identified in previous genetic studies. Further, we are likely underpowered to detect associations in many of these genes.

These results highlight the multifactorial nature of asthma risk, with both viral infection and inherited genetic variants contributing to increasing asthma risk. While the results presented for both IL33 and IL17RB are statistically significant and are potential targets for designing therapeutics and early screening, these results must be replicated in an independent study. Further work must also be done to determine if the genetic effects on asthma risk are dependent on specific treatment responses, a question requiring a much larger sample size.

## Data availability statement

The dataset presented in this article is not readily available because Norwegian law limits the sharing of sensitive data which includes genetic sequence data. Requests to access the dataset should be directed to Drs. Kari Risnes and Andrew DeWan.

## Ethics statement

The study was approved by the Regional Committee on Medical Research Ethics (REK number 2016/540). Written informed consent to participate in this study was provided by the participants’ legal guardian/next of kin.

## Author contributions

KR, IJ and AD conceived of the overall study design. IJ, AM, HD and KR led the clinical follow-up and data collection. AM performed the clinical data analysis and phenotype generation. ZD performed the genetic analyses. KR, IJ and AD supervised the work. ZD, AM, IJ, TJ, HD, KR and AD interpreted the results. ZD, AM, KR and AD drafted the initial manuscript. All authors contributed to and approved the final manuscript.
