# Supplementary material for: Type 2 cytokine genes as allergic asthma risk factors after viral bronchiolitis in early childhood
Source: Front Immunol. 2023 Jan 6;13:1054119. doi: 10.3389/fimmu.2022.1054119 (PMC9852873; doi:10.3389/fimmu.2022.1054119)
Supplement: Supplementary file 1 [file Table_1.docx]

| Table S1: Single variant results for all variants | | | | | | | |
| --- | --- | --- | --- | --- | --- | --- | --- |
| Variant^1^ | Chr^2^ | Pos^3^ | Gene^4^ | P-value^5^ | MAF^6^ | Direction^7^ | Annotation^8^ |
| NA | 2 | 112913101 | IL37 | 0.534 | 0.005 | - | intronic |
| rs3811045 | 2 | 112913726 | IL37 | 0.034 | 0.404 | + | intronic |
| NA | 2 | 112913783 | IL37 | 0.681 | 0.009 | - | intronic |
| rs1350812080 | 2 | 112913808 | IL37 | 0.836 | 0.055 | + | exonic (synonymous) |
| rs1558801106 | 2 | 112913832 | IL37 | 0.219 | 0.050 | - | exonic (synonymous) |
| rs3811047 | 2 | 112913833 | IL37 | 0.626 | 0.665 | - | exonic (synonymous) |
| NA | 2 | 112913834 | IL37 | 0.784 | 0.028 | - | exonic (synonymous) |
| NA | 2 | 112913835 | IL37 | 0.566 | 0.014 | - | exonic (synonymous) |
| rs550587376 | 2 | 112915136 | IL37 | 0.626 | 0.005 | + | intronic |
| rs2708940 | 2 | 112915313 | IL37 | 0.483 | 0.096 | + | intronic |
| rs2708943 | 2 | 112917132 | IL37 | 0.313 | 0.092 | + | exonic (synonymous) |
| rs2723183 | 2 | 112917144 | IL37 | 0.313 | 0.092 | + | exonic (synonymous) |
| rs2708944 | 2 | 112917310 | IL37 | 0.660 | 0.083 | + | intronic |
| rs2723187 | 2 | 112917692 | IL37 | 0.820 | 0.101 | + | exonic (synonymous) |
| rs2708947 | 2 | 112918642 | IL37 | 0.481 | 0.083 | + | exonic (synonymous) |
| rs2723192 | 2 | 112918804 | IL37 | 0.483 | 0.096 | + | exonic (synonymous) |
| NA | 2 | 112918864 | IL37 | 0.535 | 0.000 | NA | UTR3 |
| NA | 2 | 112918867 | IL37 | 0.535 | 0.000 | NA | UTR3 |
| rs1025690 | 3 | 53848636 | IL17RB | 0.753 | 0.422 | + | intronic |
| rs1025689 | 3 | 53849695 | IL17RB | 0.957 | 0.330 | - | exonic (synonymous) |
| NA | 3 | 53849767 | IL17RB | 0.626 | 0.005 | - | exonic (synonymous) |
| rs369284626 | 3 | 53852111 | IL17RB | 0.567 | 0.005 | + | exonic (synonymous) |
| rs3733075 | 3 | 53852885 | IL17RB | 0.056 | 0.335 | - | exonic (synonymous) |
| rs41276457 | 3 | 53853029 | IL17RB | 0.034 | 0.005 | - | intronic |
| NA | 3 | 53855230 | IL17RB | 0.568 | 0.005 | + | intronic |
| rs1432603766 | 3 | 53855242 | IL17RB | 0.684 | 0.014 | + | intronic |
| rs746036787 | 3 | 53855303 | IL17RB | 0.534 | 0.005 | - | exonic (synonymous) |
| rs2232337 | 3 | 53855341 | IL17RB | 0.604 | 0.005 | - | exonic (synonymous) |
| rs2232338 | 3 | 53855412 | IL17RB | 0.841 | 0.028 | + | intronic |
| rs191459061 | 3 | 53857002 | IL17RB | 0.534 | 0.005 | - | intronic |
| rs2232345 | 3 | 53858693 | IL17RB | 0.229 | 0.037 | - | intronic |
| rs2232346 | 3 | 53858803 | IL17RB | 0.825 | 0.041 | + | exonic (synonymous) |
| NA | 3 | 53860099 | IL17RB | 0.607 | 0.174 | + | intronic |
| rs768551820 | 3 | 53865085 | IL17RB | 0.128 | 0.014 | - | exonic (synonymous) |
| NA | 3 | 53865091 | IL17RB | 0.084 | 0.009 | - | exonic (synonymous) |
| rs1043261 | 3 | 53865249 | IL17RB | 0.369 | 0.064 | + | exonic (synonymous) |
| rs66720724 | 3 | 53865480 | IL17RB | 0.841 | 0.028 | + | UTR3 |
| rs709324 | 3 | 53865538 | IL17RB | 0.103 | 0.326 | - | UTR3 |
| rs3017 | 3 | 53865553 | IL17RB | 0.103 | 0.326 | - | UTR3 |
| rs3774626 | 3 | 53865574 | IL17RB | 0.050 | 0.032 | - | UTR3 |
| rs6798958 | 3 | 53865771 | IL17RB | 0.607 | 0.005 | + | UTR3 |
| NA | 5 | 111069914 | TSLP | 0.569 | 0.252 | - | intergenic |
| NA | 5 | 111069973 | TSLP | 0.142 | 0.005 | + | intergenic |
| rs3806932 | 5 | 111069977 | TSLP | 0.013 | 0.408 | + | intergenic |
| rs113246175 | 5 | 111070310 | TSLP | 0.604 | 0.005 | - | intergenic |
| NA | 5 | 111070329 | TSLP | 0.604 | 0.005 | - | intergenic |
| NA | 5 | 111070429 | TSLP | 0.037 | 0.023 | + | intergenic |
| rs191607411 | 5 | 111070438 | TSLP | 0.568 | 0.005 | + | intergenic |
| rs1321806970 | 5 | 111070440 | TSLP | 0.604 | 0.005 | - | intergenic |
| rs1441038237 | 5 | 111070446 | TSLP | 0.095 | 0.055 | + | intergenic |
| NA | 5 | 111070462 | TSLP | 0.437 | 0.174 | - | intergenic |
| rs560171971 | 5 | 111070531 | TSLP | 0.759 | 0.009 | - | intergenic |
| rs185194032 | 5 | 111070952 | TSLP | 0.160 | 0.009 | + | upstream |
| rs3806933 | 5 | 111071044 | TSLP | 0.013 | 0.408 | + | upstream |
| NA | 5 | 111071100 | TSLP | 0.230 | 0.009 | + | upstream |
| NA | 5 | 111071282 | TSLP | 0.762 | 0.009 | - | upstream |
| NA | 5 | 111071388 | TSLP | 0.070 | 0.046 | - | upstream |
| rs116647695 | 5 | 111071426 | TSLP | 0.182 | 0.005 | - | upstream |
| rs775210169 | 5 | 111071581 | TSLP | 0.568 | 0.005 | + | upstream |
| rs2289276 | 5 | 111071809 | TSLP | 0.222 | 0.271 | + | UTR5 |
| NA | 5 | 111072876 | TSLP | 0.634 | 0.009 | + | intronic |
| rs1561690838 | 5 | 111072939 | TSLP | 0.231 | 0.073 | + | intronic |
| rs2289277 | 5 | 111073369 | TSLP | 0.015 | 0.404 | + | intronic |
| rs1363700097 | 5 | 111073405 | TSLP | 0.279 | 0.041 | - | UTR5 |
| NA | 5 | 111073421 | TSLP | 0.030 | 0.087 | - | UTR5 |
| rs1216101828 | 5 | 111073426 | TSLP | 0.087 | 0.069 | - | UTR5 |
| rs2289278 | 5 | 111073450 | TSLP | 0.572 | 0.055 | - | UTR5 |
| rs1308038087 | 5 | 111073616 | TSLP | 0.681 | 0.005 | - | exonic (synonymous) |
| rs562538175 | 5 | 111073624 | TSLP | 0.681 | 0.005 | - | exonic (synonymous) |
| rs188293283 | 5 | 111075906 | TSLP | 0.567 | 0.005 | + | intronic |
| NA | 5 | 111076171 | TSLP | 0.684 | 0.005 | - | UTR3 |
| NA | 5 | 111076186 | TSLP | 0.324 | 0.009 | + | UTR3 |
| NA | 5 | 111076224 | TSLP | 0.642 | 0.005 | + | UTR3 |
| NA | 5 | 111076225 | TSLP | 0.642 | 0.005 | + | UTR3 |
| NA | 5 | 111076232 | TSLP | 0.642 | 0.005 | + | UTR3 |
| rs146408762 | 5 | 111076623 | TSLP | 0.774 | 0.014 | + | UTR3 |
| NA | 5 | 111076720 | TSLP | 0.607 | 0.005 | - | UTR3 |
| rs11466749 | 5 | 111076887 | TSLP | 0.040 | 0.147 | - | UTR3 |
| rs11466750 | 5 | 111077196 | TSLP | 0.001 | 0.133 | + | UTR3 |
| rs75109724 | 5 | 111077331 | TSLP | 0.773 | 0.018 | - | UTR3 |
| NA | 5 | 111077400 | TSLP | 0.567 | 0.005 | - | UTR3 |
| NA | 5 | 111077403 | TSLP | 0.567 | 0.005 | - | UTR3 |
| NA | 5 | 111077528 | TSLP | 0.732 | 0.005 | + | UTR3 |
| NA | 5 | 111077529 | TSLP | 0.761 | 0.014 | - | UTR3 |
| rs765409155 | 5 | 111077700 | TSLP | 0.567 | 0.009 | + | UTR3 |
| rs10073816 | 5 | 111077791 | TSLP | 0.030 | 0.394 | + | UTR3 |
| rs772764272 | 5 | 111078015 | TSLP | 0.604 | 0.005 | - | UTR3 |
| rs11466754 | 5 | 111078033 | TSLP | 0.038 | 0.142 | - | downstream |
| NA | 5 | 132541256 | IL5 | 0.837 | 0.041 | - | downstream |
| rs1172454363 | 5 | 132541264 | IL5 | 0.840 | 0.032 | + | downstream |
| NA | 5 | 132541281 | IL5 | 0.106 | 0.005 | - | downstream |
| rs775344061 | 5 | 132541887 | IL5 | 0.534 | 0.005 | - | exonic (synonymous) |
| rs771362124 | 5 | 132542096 | IL5 | 0.535 | 0.005 | - | exonic (synonymous) |
| rs2069822 | 5 | 132543190 | IL5 | 0.346 | 0.014 | + | intronic |
| rs149977915 | 5 | 132543361 | IL5 | 0.067 | 0.005 | + | exonic (synonymous) |
| NA | 5 | 132543512 | IL5 | 0.568 | 0.005 | - | UTR5 |
| rs894926573 | 5 | 132658040 | IL13 | 0.534 | 0.005 | - | intronic |
| rs1162221213 | 5 | 132658344 | IL13 | 0.732 | 0.005 | + | exonic (synonymous) |
| NA | 5 | 132658354 | IL13 | 0.732 | 0.005 | + | exonic (synonymous) |
| rs201923060 | 5 | 132659508 | IL13 | 0.141 | 0.009 | - | intronic |
| rs1295686 | 5 | 132660151 | IL13 | 0.431 | 0.225 | - | intronic |
| NA | 5 | 132660164 | IL13 | 0.361 | 0.023 | + | intronic |
| rs20541 | 5 | 132660272 | IL13 | 0.461 | 0.220 | - | exonic (synonymous) |
| NA | 5 | 132660482 | IL13 | 0.598 | 0.009 | - | UTR3 |
| rs115325936 | 5 | 132660597 | IL13 | 0.567 | 0.005 | - | UTR3 |
| NA | 5 | 132660851 | IL13 | 0.769 | 0.110 | + | UTR3 |
| rs847 | 5 | 132660977 | IL13 | 0.543 | 0.216 | - | UTR3 |
| rs143032763 | 5 | 132661060 | IL13 | 0.535 | 0.005 | + | UTR3 |
| rs2070874 | 5 | 132674018 | IL4 | 0.698 | 0.174 | - | UTR5 |
| rs765889733 | 5 | 132674078 | IL4 | 0.220 | 0.009 | - | exonic (synonymous) |
| rs56141757 | 5 | 132674524 | IL4 | 0.627 | 0.009 | + | intronic |
| rs71645916 | 5 | 132679908 | IL4 | 0.106 | 0.005 | - | ncRNA_exonic |
| rs2243289 | 5 | 132682440 | IL4 | 0.698 | 0.174 | - | ncRNA_intronic |
| rs2243290 | 5 | 132682477 | IL4 | 0.606 | 0.179 | - | ncRNA_exonic |
| rs555998964 | 9 | 6215178 | IL33 | 0.229 | 0.005 | + | UTR5 |
| rs746808236 | 9 | 6215231 | IL33 | 0.608 | 0.005 | - | intronic |
| rs1431798023 | 9 | 6215727 | IL33 | 0.125 | 0.005 | - | intronic |
| rs17498168 | 9 | 6237186 | IL33 | 0.211 | 0.028 | - | intronic |
| NA | 9 | 6250936 | IL33 | 0.496 | 0.037 | + | intronic |
| rs1317230 | 9 | 6251012 | IL33 | 0.205 | 0.321 | - | intronic |
| rs138828591 | 9 | 6251299 | IL33 | 1.000 | 0.041 | + | intronic |
| rs73398552 | 9 | 6252689 | IL33 | 0.039 | 0.041 | - | intronic |
| rs149045797 | 9 | 6252690 | IL33 | 0.535 | 0.005 | + | intronic |
| rs1397714619 | 9 | 6252710 | IL33 | 0.142 | 0.005 | + | intronic |
| NA | 9 | 6252720 | IL33 | 0.142 | 0.005 | + | intronic |
| rs10975519 | 9 | 6253571 | IL33 | 0.861 | 0.372 | - | exonic (synonymous) |
| rs562550122 | 9 | 6253690 | IL33 | 0.068 | 0.005 | - | intronic |
| rs10975520 | 9 | 6253710 | IL33 | 0.660 | 0.362 | - | intronic |
| NA | 9 | 6255774 | IL33 | 0.626 | 0.005 | + | intronic |
| NA | 9 | 6255781 | IL33 | 0.626 | 0.005 | + | intronic |
| rs12336076 | 9 | 6255789 | IL33 | 0.602 | 0.326 | - | intronic |
| NA | 9 | 6255807 | IL33 | 0.600 | 0.243 | - | intronic |
| NA | 9 | 6255808 | IL33 | 0.785 | 0.028 | - | intronic |
| NA | 9 | 6255814 | IL33 | 0.784 | 0.110 | - | . |
| rs1013300156 | 9 | 6255821 | IL33 | 0.819 | 0.133 | - | intronic |
| NA | 9 | 6255826 | IL33 | 0.870 | 0.101 | + | intronic |
| rs1332290 | 9 | 6255881 | IL33 | 0.403 | 0.422 | - | intronic |
| rs146597587 | 9 | 6255967 | IL33 | 0.535 | 0.005 | + | splicing |
| rs1240440599 | 9 | 6256010 | IL33 | 0.732 | 0.005 | + | exonic (synonymous) |
| NA | 9 | 6256013 | IL33 | 0.732 | 0.005 | + | exonic (synonymous) |
| rs35375147 | 9 | 6256078 | IL33 | 0.344 | 0.014 | - | exonic (synonymous) |
| rs1048274 | 9 | 6256292 | IL33 | 0.835 | 0.376 | - | UTR3 |
| NA | 9 | 6256471 | IL33 | 0.542 | 0.050 | + | UTR3 |
| NA | 9 | 6256476 | IL33 | 0.789 | 0.023 | - | UTR3 |
| rs55726619 | 9 | 6256678 | IL33 | 0.039 | 0.041 | - | UTR3 |
| NA | 9 | 6256741 | IL33 | 0.035 | 0.005 | + | UTR3 |
| rs12000491 | 9 | 6257367 | IL33 | 0.039 | 0.041 | - | UTR3 |
| rs189961633 | 9 | 6257571 | IL33 | 0.787 | 0.009 | - | UTR3 |
| NA | 9 | 6257597 | IL33 | 0.099 | 0.014 | + | UTR3 |
| rs553100713 | 9 | 6257606 | IL33 | 0.068 | 0.009 | + | UTR3 |
| NA | 9 | 6257718 | IL33 | 0.607 | 0.005 | - | UTR3 |
| rs73398574 | 9 | 6257724 | IL33 | 0.039 | 0.041 | - | UTR3 |
| rs7145531 | 14 | 23372808 | IL25 | 0.739 | 0.275 | + | upstream |
| rs7145551 | 14 | 23372831 | IL25 | 0.739 | 0.275 | + | UTR5 |
| rs144496239 | 14 | 23372938 | IL25 | 0.684 | 0.005 | - | UTR5 |
| NA | 14 | 23373072 | IL25 | 0.535 | 0.000 | NA | UTR5 |
| rs11465506 | 14 | 23373452 | IL25 | 0.690 | 0.009 | + | intronic |
| rs1341710950 | 14 | 23375528 | IL25 | 0.568 | 0.005 | - | intronic |
| rs145160878 | 14 | 23375644 | IL25 | 0.535 | 0.005 | + | exonic (synonymous) |
| rs973711361 | 14 | 23375691 | IL25 | 0.568 | 0.005 | - | exonic (synonymous) |
| rs1124053 | 14 | 23375770 | IL25 | 0.993 | 0.303 | + | exonic (synonymous) |
| rs777553594 | 14 | 23375773 | IL25 | 0.635 | 0.009 | - | exonic (synonymous) |
| rs201592081 | 14 | 23375883 | IL25 | 0.607 | 0.005 | - | UTR3 |
| NA | 14 | 23375980 | IL25 | 0.569 | 0.005 | - | UTR3 |
| NA | 14 | 23376003 | IL25 | 0.680 | 0.014 | + | UTR3 |
| rs1262341869 | 14 | 23376013 | IL25 | 0.568 | 0.005 | - | UTR3 |
| rs1245712878 | 14 | 23376020 | IL25 | 0.351 | 0.023 | - | UTR3 |
| rs3811178 | 14 | 23376035 | IL25 | 0.954 | 0.289 | + | UTR3 |
| rs189524544 | 14 | 23376081 | IL25 | 0.569 | 0.005 | - | UTR3 |
| rs11465521 | 14 | 23376145 | IL25 | 0.513 | 0.037 | + | UTR3 |
| rs924540235 | 14 | 23376212 | IL25 | 0.566 | 0.005 | - | UTR3 |
| rs151218732 | X | 1196817 | CRLF2 | 0.849 | 0.050 | + | exonic (synonymous) |
| rs374608816 | X | 1196912 | CRLF2 | 0.534 | 0.005 | + | intronic |
| rs187497694 | X | 1196921 | CRLF2 | 0.361 | 0.018 | - | intronic |
| rs199794164 | X | 1198676 | CRLF2 | 0.106 | 0.005 | - | exonic (synonymous) |
| rs1341440674 | X | 1198713 | CRLF2 | 0.627 | 0.009 | + | exonic (synonymous) |
| rs1292358347 | X | 1198716 | CRLF2 | 0.104 | 0.005 | + | exonic (synonymous) |
| NA | X | 1198717 | CRLF2 | 0.373 | 0.028 | - | exonic (synonymous) |
| rs768515067 | X | 1198718 | CRLF2 | 0.534 | 0.005 | + | exonic (synonymous) |
| rs776574225 | X | 1198720 | CRLF2 | 0.662 | 0.050 | + | exonic (synonymous) |
| rs1462327387 | X | 1198721 | CRLF2 | 0.798 | 0.014 | + | exonic (synonymous) |
| rs761735541 | X | 1198722 | CRLF2 | 0.569 | 0.005 | - | exonic (synonymous) |
| NA | X | 1198724 | CRLF2 | 0.642 | 0.005 | + | exonic (synonymous) |
| rs1360344640 | X | 1198726 | CRLF2 | 0.361 | 0.018 | + | splicing |
| rs765072848 | X | 1198727 | CRLF2 | 0.535 | 0.005 | + | intronic |
| rs1456401562 | X | 1198728 | CRLF2 | 0.500 | 0.005 | + | intronic |
| rs1555888068 | X | 1198731 | CRLF2 | 0.006 | 0.183 | + | intronic |
| rs138899368 | X | 1202479 | CRLF2 | 0.498 | 0.032 | + | exonic (synonymous) |
| rs147809511 | X | 1208762 | CRLF2 | 0.818 | 0.018 | + | intronic |
| rs374312439 | X | 1212581 | CRLF2 | 0.080 | 0.028 | + | exonic (synonymous) |
| rs140859855 | X | 1212602 | CRLF2 | 0.229 | 0.234 | - | exonic (synonymous) |
| NA | X | 1212634 | CRLF2 | 0.534 | 0.005 | - | exonic (synonymous) |
| NA | X | 1212651 | CRLF2 | 0.534 | 0.005 | - | UTR5 |
| rs150166261 | X | 1212677 | CRLF2 | 0.128 | 0.018 | - | UTR5 |
| NA | X | 1212694 | CRLF2 | 0.642 | 0.005 | + | UTR5 |
| NA | X | 1212710 | CRLF2 | 0.535 | 0.005 | + | UTR5 |
| NA | X | 1212712 | CRLF2 | 0.535 | 0.005 | + | UTR5 |

^1^Variant identification number (rsID), if assigned

^2^Chromosome

^3^Base pair position on the chromosome, in hg38 coordinates

^4^Gene in which the variant was identified

^5^P-value from the SKAT test

^6^Minor Allele Frequency

^7^Direction of effect estimated using logistic regression, where “+” represents a variant with a minor allele that has a higher frequency among asthmatic children and “-” represents a variant with a minor allele that has a lower frequency among asthmatic children.

^8^Functional annotation according to refGene; UTR3 = 3’ untranslated region; UTR5 = 5’ untranslated region
